# *QuickStats:* Birth Rates* for Teens Aged 15–19 Years, by State — National Vital Statistics System, United States, 2018

**DOI:** 10.15585/mmwr.mm6844a5

**Published:** 2019-11-08

**Figure:**
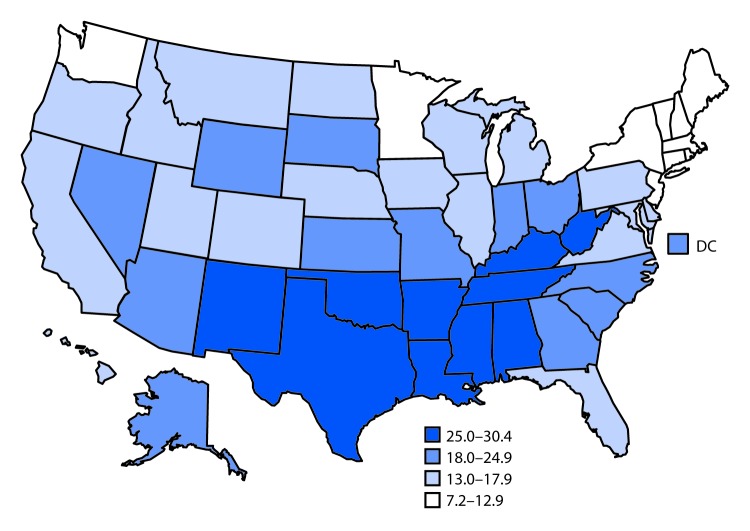
In 2018, the U.S. birth rate for teens aged 15–19 years was 17.4 births per 1,000 females, with rates generally lower in the Northeast and higher across the southern states. Teen birth rates ranged from 7.2 in Massachusetts, 8.0 in New Hampshire, 8.3 in Connecticut, and 8.8 in Vermont to rates of 30.4 in Arkansas, 27.8 in Mississippi, 27.5 in Louisiana, 27.3 in Kentucky, and 27.2 in Oklahoma.

**Source:** National Vital Statistics System. Birth data, 2018. https://www.cdc.gov/nchs/nvss/births.htm.

